# P-904. Spinal Arachnoiditis and Syringomyelia in Coccidioidomycosis: A Case Series of 53 Adult Patients

**DOI:** 10.1093/ofid/ofae631.1095

**Published:** 2025-01-29

**Authors:** Bianca Torres, Danish Khalid, Chelsea Dunn, Rupam Sharma, Lovedip Kooner, Michael Valdez, Shikha Mishra, Carlos M D’Assumpcao, Royce H Johnson, Rasha Kuran

**Affiliations:** Valley Fever Institute at Kern Medical, Bakersfield, California; Valley Fever Institute at Kern Medical, Bakersfield, California; Valley Fever Institute at Kern Medical, Bakersfield, California; Kern Medical Center, Bakersfield, California; Valley Fever Institute at Kern Medical, Bakersfield, California; Kern Medical, Bakersfield, California; Kern Medical-UCLA, Bakersfield, California; Kern Medical, Bakersfield, CA, Bakersfield, California; Kern Medical Center, Bakersfield, California; Kern Medical Center, Bakersfield, California

## Abstract

**Background:**

Central nervous system (CNS) involvement with coccidioidomycosis (cocci) is a serious infection that is universally fatal if not treated. Prior to the advent of magnetic resonance imaging (MRI), very few reports describe spinal cord involvement and autopsies often omitted spinal cord examination. Accurate and anatomic localization of areas affected by CNS cocci can be challenging due to mental status changes and the presence of brain abnormalities. Here, we describe radiologic and clinical characteristics of 53 cases of CNS cocci who had spinal MRI imaging performed.

**Methods:**

This retrospective case series reviewed 53 adult patients at the Valley Fever Institute in Bakersfield, California. Patients had confirmed CNS cocci and MRI of at least one segment of the spinal image with and without contrast. A waiver of consent was submitted, and approval was obtained by Kern Medical’s Institutional Review Board. ICD 9 and ICD 10 codes were used to query electronic health record and cross referenced with imaging. Each record reviewed required inclusion criteria of age above 18 years, cerebrospinal fluid (CSF) abnormalities compatible with chronic meningitis and one of the following: positive CSF IgG antibody, CSF complement fixation (CF) or growth of Coccidioides on CSF culture. MRI Imaging with and without contrast of at least one of the following: cervical, thoracic or lumbar spine.

**Results:**

77% of the patients were Latinx, 13% African American, 4% Asian and 6% Caucasian. Males make up the majority at 72%. 37% had intrathecal (IT) amphotericin (ABD) injections. Spinal cord abnormalities can be asymptomatic with the most common symptom of back pain followed by radiculopathy. Abnormal findings included leptomeningeal enhancement, arachnoid cysts, masses or adhesions, syringomyelia, myelitis, cord edema, myelomalcia, flattened cord, nerve root "clumping" or solid cauda equina.

**Conclusion:**

This study suggests the benefit of obtaining neuro axis imaging with and without contrast on all new cases of Coccidioidal meningitis. Spinal cord involvement with CNS cocci is common and contributes to significant morbidity. Contrary to common belief, our study shows that spinal arachnoiditis occurs in patients who were never treated with IT ABD. This may suggest that IT ABD may not cause arachnoiditis.

**Disclosures:**

**All Authors**: No reported disclosures

